# EQUATOR reporting guideline use and editorial enforcement in four medical specialties: a cross-sectional study of 20 high-impact journals

**DOI:** 10.1186/s12874-026-02901-5

**Published:** 2026-06-05

**Authors:** Antonio Cisneros-Barrios, Pamela Hernández-Delgado, Paloma Gradilla-Magaña, Ismael Gutierrez-Jimenez, Cesar Ramos-Remus

**Affiliations:** 1 Instituto Mexicano del Seguro Social, Division de Oftalmologia, Hospital de Especialidades, Centro Medico Nacional Siglo XXI, Cuahtemoc 330, Ciudad de Mexico, 06720 Mexico; 2 Instituto de Oftalmología FAP Conde de Valenciana IAP, Chimalpopoca 14, Ciudad de Mexico, 06800 Mexico; 3 Centro Universitario de Ciencias de la Salud, Universidad de Guadalajara, Sierra Mojada 950, Guadalajara, Jalisco 44340 Mexico; 4 Unidad de Investigación en Enfermedades Cronico Degenerativas, Colomos 2292, Guadalajara, Jalisco 44620 Mexico

**Keywords:** Ophthalmology, Editorial Policies, Research, Publishing, Reporting Guidelines, EQUATOR Network

## Abstract

**Background:**

EQUATOR (Enhancing the QUAlity and Transparency Of Health Research) reporting guidelines aim to improve transparency and completeness in biomedical research; however, their adoption remains inconsistent across medical specialties. We quantified declared reporting guideline use in high-impact ophthalmology journals and assessed whether editorial policies are associated with their implementation across specialties.

**Methods:**

We conducted a cross-sectional study of original research articles published in 2024 across five first-quartile ophthalmology journals. Reporting guideline use was identified through systematic full-text searches. An “advisable use rate” was defined as the proportion of articles declaring guideline use among those for which use was methodologically appropriate. Journal enforcement policies were characterized through analysis of instructions for authors. Guideline use in ophthalmology was contextualized through comparison with previously published datasets from orthopedics, rheumatology, and infectious diseases.

**Results:**

Among 794 original research articles, reporting guidelines were deemed advisable for 672 (84.6%). Of these, 192 articles (28.6%) declared guideline use. Advisable use rates ranged from 5% to 120% across journals. STROBE was the most frequently applicable guideline but was declared in only 27% of eligible articles. Ophthalmology guideline use exceeded orthopedics (10.2%), rheumatology (7.2%), and infectious diseases (6.3%). Although enforcement policies varied across journals, no significant correlation was observed between enforcement level and guideline use (Kendall’s τ = 0.24, *p* > 0.05).

**Conclusions:**

Despite higher adoption relative to other specialties, reporting guideline use in ophthalmology remains limited. Substantial inter-journal variability and the absence of an association between enforcement policies and use highlight opportunities to better align editorial expectations with reporting practices.

## Introduction

Incomplete or inaccurate reporting undermines the foundation of evidence-based medicine. Systematic deficiencies in how studies are reported compromise the interpretation, reproducibility, and clinical applicability of published research [1], representing avoidable waste in the production and dissemination of scientific evidence [2].

To address these deficiencies, structured reporting guidelines were developed to improve the quality, completeness, and transparency of how research is reported, rather than the methodological rigor of the underlying studies, by providing standardized frameworks for presenting research findings [3]. The Consolidated Standards of Reporting Trials (CONSORT) were the first to establish standards for reporting randomized clinical trials [4], followed by guidelines for other study designs, including STROBE for observational studies and PRISMA for systematic reviews [5, 6].

In 2006, these initiatives were unified under the EQUATOR (Enhancing the QUAlity and Transparency Of Health Research) Network, a global effort aimed at promoting transparent and accurate reporting across diverse research methodologies [7]. Since then, reporting guidelines have been widely disseminated, freely accessible, and formally endorsed by major biomedical journals and publishers.

Despite this widespread availability and endorsement, multiple studies have demonstrated limited adoption of reporting guidelines across medical specialties. In rheumatology and infectious diseases, guidelines were declared in only 7.2% and 6.3% of manuscripts for which their use was advisable, respectively [8, 9]. The persistence of this pattern suggests that factors beyond author awareness—such as editorial context and enforcement practices—may influence guideline implementation.

Ophthalmology presents unique challenges for standardized reporting. Visual acuity outcomes require precise notation systems, imaging modalities such as optical coherence tomography demand detailed acquisition and interpretation protocols, and surgical interventions necessitate comprehensive descriptions of techniques and instrumentation. Despite these complexities, no systematic assessment of EQUATOR reporting guideline use in ophthalmology has been conducted, representing a critical gap in understanding reporting practices within the specialty.

Accordingly, the present study assessed the declared use of EQUATOR reporting guidelines in original research articles published during 2024 in five high-impact ophthalmology journals. In addition, we examined journal-level editorial policies regarding reporting guidelines and explored whether differences in enforcement were associated with observed patterns of guideline use. To contextualize these findings, guideline adoption in ophthalmology was compared with previously published data from orthopedics, rheumatology, and infectious diseases.

## Materials and methods

### Study design

This was a cross-sectional, audit-type study of original research articles published during 2024 in five specialized ophthalmology journals.

### Journal selection

From the 36 journals ranked in the first quartile (Q1) of the Scimago Journal Rank (SJR) for ophthalmology [10], we selected five journals based on the following criteria: (1) primary focus on ophthalmology, (2) highest impact factors within Q1, and (3) publication of at least 20% original research articles in 2024. This threshold was established to exclude journals that predominantly publish review articles, as reporting guidelines apply specifically to original research. We limited our sample to five journals to ensure the feasibility of manual, article-by-article assessment while capturing a representative sample of high-impact ophthalmology literature. We evaluated 18 core EQUATOR reporting guidelines, which represent the most widely applicable guidelines across common study designs. The selected journals were Ophthalmology, JAMA Ophthalmology, American Journal of Ophthalmology, British Journal of Ophthalmology, and The Ocular Surface.

### Article selection

Articles were initially identified as original research based on their classification in the journal’s table of contents and their indexing as original articles in the Web of Science Core Collection. All identified articles were then downloaded as Portable Document Files (PDFs) from an institutional subscription and manually reviewed to confirm eligibility.

Editorials, narrative reviews, letters to the editor, commentaries, and short communications were excluded. Inclusion was based on journal classification as original research rather than study design alone. Therefore, systematic reviews were included when classified as original research by the journal, and corresponding reporting guidelines (e.g., PRISMA) were evaluated as part of the EQUATOR framework.

### Assessment of reporting guidelines use

Articles were downloaded as PDFs by one reviewer (ACB), who conducted an initial systematic search using Adobe Acrobat Reader for the following terms: (1) the acronyms of the 18 EQUATOR guidelines evaluated (CONSORT, STROBE, PRISMA, MOOSE, SPIRIT, STARD, TRIPOD, CARE, STREGA, ARRIVE, RECORD, COREQ, AGREE, CHERRIES, SQUIRE, CHEERS, STARI, CREDES; full names available at www.equator-network.org), (2) the term “reporting guideline,” and (3) the term “EQUATOR.” A guideline was considered “used” if its acronym or full name appeared in the manuscript body, excluding the reference list.

A second reviewer (CRR) independently verified that all included articles represented original research and assessed whether the use of a reporting guideline was advisable for each article based on its study design and methodology. This assessment involved matching study characteristics with the scope and intended applications of available EQUATOR guidelines. An “advisable use rate” was calculated as the proportion of articles that declared using a specific guideline among those for which the guideline was deemed advisable.

### Cross-specialty contextual comparison

To contextualize our findings within the broader medical literature, we used previously published datasets from three medical specialties—orthopedics, rheumatology, and infectious diseases—to provide a contextual comparison with the ophthalmology data. These data were obtained from prior studies conducted by members of our research team using identical methodology [8, 9, 11].

The orthopedics dataset included 5 journals (The Journal of Arthroplasty, Arthroscopy, The Journal of Bone and Joint Surgery, Osteoarthritis and Cartilage, and Journal of Bone and Mineral Research), comprising 1,495 articles published in 2019 [11]. The rheumatology dataset encompassed 5 journals (Annals of the Rheumatic Diseases, Arthritis & Rheumatology, Arthritis Care and Research, Rheumatology, and The Journal of Rheumatology), including 895 articles from 2019 [8]. The infectious diseases dataset included 5 journals (Lancet Infectious Diseases, Lancet HIV, Journal of Infectious Diseases, Journal of Infection, and Clinical Infectious Diseases), comprising 1,251 articles from 2019 [9].

For the enforcement level analysis, we combined data from all 20 journals (5 from each specialty) to explore the relationship between editorial policies and reporting guideline implementation across medical specialties.

### Statistical analysis

Categorical variables are presented as frequencies and percentages with 95% confidence intervals. An “advisable use rate” was calculated as the proportion of articles that declared using a specific guideline among those for which the guideline was deemed advisable.

We characterized the enforcement policies of all 20 journals (5 from ophthalmology and 15 from the comparison specialties) based on their instructions for authors accessed from each journal’s website during October 2025, using a classification system that considered the strength of language used, verification mechanisms, and consequences for non-compliance. Both Kendall’s tau-b and Spearman’s rho were calculated to assess the correlation between enforcement level and guideline use rate. Statistical analyses were performed using SPSS (version 28). A P-value < 0.05 was considered statistically significant.

## Results

A total of 1,454 articles were published in the five selected journals during 2024. After excluding non-research articles, 794 (54.6%) were classified as original research and underwent full assessment. The use of a reporting guideline was deemed advisable for 672 (84.6%; 95% CI, 82%-87%) of these articles. Reporting guidelines were used in 192 articles, representing 24.2% (95% CI, 21%-27%) of all reviewed articles and 28.6% (95% CI, 25%-32%) of articles for which guidelines were advisable.

Table 1 presents the frequency distribution of reporting guideline use and the advisable use rate by journal. JAMA Ophthalmology demonstrated the highest advisable use rate (120%; 95% CI, 94%-146%), while the other four journals showed substantially lower rates, ranging from 5% (95% CI, 1%-16%) in The Ocular Surface to 25% (95% CI, 17%-35%) in Ophthalmology. Although STROBE was the most frequently advisable guideline across all journals, it was declared in only 87 of 317 articles for which it was deemed appropriate (27%). The ARRIVE guideline, applicable to animal research, was used in only 2 of 24 advisable articles (8%).

**Table 1:** Frequency distribution of actual and advisable use of reporting guidelines and advisable use rate by selected ophthalmology journals

	Ophthalmology	JAMA Ophthalmol	Am J Ophthalmol	Br J Ophthalmol	Ocul Surf
Assessed articles, *n*	122	86	293	226	67
Advisable articles for reporting guidelines, n (%)	102 (83.6)	70 (81.3)	264 (90.1)	185 (81.8)	51 (76.1)
Use of any reporting guidelines, n (AR*) [95%CI]	26 (0.25) [17 to 35]	84 (1.2) [94 to 100]	51 (0.17) [14 to 24]	28 (0.15) [10 to 21]	3 (0.05) [1 to 16]

Reporting Guidelines	Actual Use/Advisable Use (Advisable Rate*)				
CONSORT^**^	7 / 31 (0.22)	15/ 12 (1.25)	5 / 31 (0.16)	12 / 28 (0.42)	1 / 7 (0.14)
STROBE^#^	11 / 41 (0.26)	53 / 31 (1.70)	19 / 137 (0.13)	4 / 98 (0.04)	0 / 10 (0)
PRISMA^$^ or MOOSE^&^	8 / 11 (0.72)	4 / 3 (1.33)	25 / 26 (0.96)	6 / 6 (1)	0 / 0
SPIRIT^+^	0 / 1 (0)	0 / 0	0 / 0	0 / 1 (0)	0 / 0
STARD^@^	0 / 0	5 / 1 (5)	1 / 22 (0.04)	3 / 11 (0.27)	0 / 2 (0)
TRIPOD°	0 / 3 (0)	1 / 0	0 / 8 (0)	1 / 11 (0.09)	0 / 1 (0)
CARE^ø^	0 / 2 (0)	1 / 7 (0.14)	0 / 0	1 / 1 (1)	0 / 3 (0)
STREGAª	0 / 2 (0)	0 / 1 (0)	0 / 14	0 / 6 (0)	0 / 0
ARRIVE^§^	0 / 0 (0)	0 / 0	0 / 0	0 / 0	2 / 24 (0.08)
RECORD^¤^:	0 / 9 (0)	1 / 11 (0.09)	1 / 30 (0.03)	0/ 21 (0)	0 / 1 (0)
COREQ^ɸ^:	0 / 0	0 / 0	0 / 0	0 /0	0 / 0
AGREE ***	0 / 0	0 / 1 (0)	0 / 1 (0)	0 / 1 (0)	0/0
CHERRIES^œ^	0 / 0	0 /0	0 / 1 (0)	0 / 1 (0)	0 / 3 (0)
SQUIRE^¶^	0 / 1 (0)	2 / 2 (1)	0 / 0	0 / 0	0 / 0
CHEERS^✽^	0 / 1 (0)	1 / 1 (1)	0 / 1 (0)	0 / 1 (0)	0 / 0
STARI°°	0 / 0	0/0	0 / 0	1 / 0	0 / 0
CREDES^⧫^	0 / 0	1 / 0	0 / 0	0 / 0	0 / 0

*AR Advisable Rate; proportion related to the advisable articles

**CONSORT: CONsolidated Standards of Reporting Trials

# STROBE: Strengthening the Reporting of Observational Studies in Epidemiology

$ PRISMA: Preferred Reporting Items for Systematic Reviews and Meta-Analysis

& MOOSE: Meta-analysis Of Observational Studies in Epidemiology

+ SPIRIT: Standard Protocol Items: Recommendations for Interventional Trials

@ STARD: Standards for Reporting Diagnostic accuracy studies

° TRIPOD: Transparent reporting of a multivariable prediction model for individual prognosis or diagnosis

Ø CARE: CAse REports

ª STREGA: STrengthening the REporting of Genetic Association Studies

§ ARRIVE: Animal Research: Reporting of In Vivo Experiments

¤ RECORD: REporting of studies Conducted using Observational Routinely-collected Data

ɸ COREQ: Consolidated criteria for reporting qualitative research

***AGREE: Reporting Guideline Checklist

œ CHERRIES: Checklist for Reporting Results of Internet E-Surveys

¶ SQUIRE Standards for QUalityImprovement Reporting Excellence

✽ CHEERS Consolidated Health Economic Evaluation Reporting Standards

°° STARI Standards for Reporting Implementation Studieso

⧫ CREDES Conducting and Eporting DElphi Studies

Table 2 compares reporting guideline use in ophthalmology with three previously studied specialties [8, 9, 11]. Ophthalmology demonstrated the highest rate of guideline use (28.6%), followed by orthopedics (10.2%), rheumatology (7.2%), and infectious diseases (6.3%) [8, 9, 11]. Despite being the most frequently advisable guideline across all specialties, STROBE was declared in only 7.1% of articles for which it was appropriate. In contrast, CONSORT showed higher utilization (25.3%), while ARRIVE remained underutilized (5.1%).

**Table 2:** Distribution of the use of reporting guidelines among four specialties

	Ophthalmology	Orthopedic	Infectious diseases	Rheumatology
Research articles to assess, *n*	794	1495	1251	895
Advisable articles for reporting guidelines, n (%) [95%CI]	672 (84) [82 to 87]	1273 (83) [83 to 87]	937 (74) [72 to 77]	693 (77) [75 to 80]
Use of any reporting guidelines, n (AR*)	192 (0.28)	130 (0.10)	59 (0.06)	50 (0.07)

Reporting Guidelines**	Actual Use /Advisable Use (Advisable Rate*)			
CONSORT^**^	40 / 109 (0.36)	61/110 (0.55)	5 /174 (0.02)	14/82 (0.17)
STROBE^#^	87 / 317 (0.27)	23/714 (0.03)	9 / 416 (0.02)	9/355 (0.02)
PRISMA or MOOSE^$^	43 / 46 (0.93)	32/40 (0.8)	36 / 58 (0.62)	17/24 (0.7)
ARRIVE^§^	2 / 26 (0.07)	7/ 108 (0.06)	1 / 60 (0.1)	2/40 (0.05)

*AR Advisable Rate; proportion related to the advisable articles

**Refer to Table 1 for the full names of the reporting guidelines

Table 3 presents the enforcement levels and guideline use rates across 20 journals from ophthalmology and the three comparison specialties [8, 9, 11]. Enforcement varied substantially across journals, with most demonstrating weak to moderate enforcement policies. Only three journals (15%) achieved the highest enforcement level (Level 6): American Journal of Ophthalmology, JAMA Ophthalmology, and Rheumatology. Despite this variation in enforcement policies, no statistically significant correlation was found between enforcement level and guideline use rate (Kendall’s τ = 0.24; Spearman’s ρ = 0.29; both *p* > 0.05).**.

**Table 3:** Enforcement level and reporting guideline use across journals in four medical specialties

Journal	Specialty	Enforcement Level	Key Enforcement Features	AR [95% CI]*
Ophthalmology	Ophthalmology	4	Strongly endorses CONSORT	0.25 [17–35]
JAMA Ophthalmol	Ophthalmology	6	Mandatory checklist, peer review verification	1.2 [94–100]
Am J Ophthalmol	Ophthalmology	6	Mandatory checklist, affects publication	0.17 [14–24]
Br J Ophthalmol	Ophthalmology	5	Required (BMJ policy)	0.15 [10–21]
Ocul Surf	Ophthalmology	2	ICMJE^@^ reference only	0.05 [1–16]
J Arthroplasty	Orthopedics	5	Required, editorial consideration	0.09 [7–12]
Arthroscopy	Orthopedics	4	Strongly encourages CONSORT	0.08 [5–12]
J Bone Joint Surg Am	Orthopedics	2	ICMJE reference only	0.18 [12–24]
Osteoarthritis Cartilage	Orthopedics	3	Advocates use	0.12 [6–20]
J Bone Miner Res	Orthopedics	5	Required, affects editorial decision	0.10 [4–13]
Lancet Infect Dis	Infectious Diseases	5	Required (Lancet policy)	0.12 [7–24]
Lancet HIV	Infectious Diseases	5	Required (Lancet policy)	0.07 [1–20]
J Infect Dis	Infectious Diseases	4	Should consult guidelines	0.06 [3–10]
J Infect	Infectious Diseases	2	ICMJE reference only	0.12 [6–21]
Clin Infect Dis	Infectious Diseases	2	ICMJE reference only	0.03 [2–6]
Ann Rheum Dis	Rheumatology	2	ICMJE reference only	0.09 [5–16]
Arthritis Rheumatol	Rheumatology	5	Required for peer review	0.04 [1–9]
Arthritis Care Res	Rheumatology	5	Required for peer review	0.10 [6–16]
Rheumatology	Rheumatology	6	Mandatory, available upon request	0.07 [4–13]
J Rheumatol	Rheumatology	2	ICMJE reference only	0.03 [0–7]

*AR indicates advisable use rate

**95% confidence interval

@ICMJE, International Committee of Medical Journal Editors

Enforcement levels were classified on a 6-point scale based on journal instructions for authors:

Level 6: Mandatory checklist submission with explicit verification and consequences

Level 5: Required with implicit or explicit verification mechanisms

Level 4: Strongly recommended or endorsed

Level 3: Advocated or suggested

Level 2: Reference to ICMJE or EQUATOR guidelines only

Level 1: Not mentioned (not present in this sample)

The Advisable Use Rate (AR) represents the proportion of articles that declared using a reporting guideline among those for which use was deemed advisable based on study design. Values > 1.0 indicate that authors applied guidelines beyond strict requirements

## Discussion

Approximately one in four ophthalmology articles for which reporting guidelines were advisable declared their use (28.6%). While this surpasses rates in orthopedics (10.2%), rheumatology (7.2%), and infectious diseases (6.3%), it represents substantial underutilization in high-impact journals that explicitly endorse guideline use. The persistence of this pattern suggests that factors beyond awareness may be influencing adoption.

Our findings reveal a fundamental disconnect in scientific publishing: journals require reporting guidelines in author instructions, yet the majority of authors do not use them. This pattern cannot be explained by lack of awareness after decades of dissemination and free accessibility [12]. Instead, examination of enforcement mechanisms suggests a more plausible explanation. Among 20 journals across four specialties, 85% mention reporting guidelines in author instructions, yet only 15% implement meaningful enforcement through verification mechanisms, defined consequences, or integration into peer review. This creates a gap between stated policy and practice: authors are instructed to follow guidelines, yet manuscripts without them face no barriers to publication. This inconsistency may signal that guideline use, despite being “required,” remains optional in practice.

Guideline use varied 24-fold across journals (5% to 120%), reflecting differing editorial approaches. Journals implementing mandatory checklist submission and verification achieved higher use rates (up to 120%), while those with minimal enforcement showed substantially lower rates (as low as 5%). However, no significant correlation was found between enforcement level and guideline use across all 20 journals (Kendall’s τ = 0.24, *p* > 0.05), suggesting that intermediate enforcement approaches yield inconsistent results. Two editorial strategies appear internally coherent: rigorous enforcement with verification mechanisms, and minimal enforcement where guidelines function as recommendations. The disconnect arises primarily among journals employing moderate enforcement that states expectations without operationalizing verification within editorial workflows.

The persistent gap between endorsement and use raises a fundamental question: Do reporting guidelines improve research quality beyond completeness? Evidence is mixed. Some studies document improvements in reporting completeness, particularly for CONSORT in randomized trials [13–15] and when guidelines are integrated into peer review [16, 17]. However, others find no significant improvement [18, 19]. Critically, reporting completeness does not equate to methodological rigor, as Logullo et al. emphasize, reporting checklists guide writing, not research design [20]. This disconnect between reporting transparency and methodological quality may explain the low adoption observed, as authors may perceive guidelines as administrative requirements rather than tools that strengthen research.

These findings have important implications for editorial policy. Our results suggest two approaches that align policy with implementation. First, systematic enforcement through mandatory checklist submission, editorial verification, and integration into manuscript decisions, as demonstrated by journals with the highest enforcement levels. Second, explicit acknowledgment that guidelines represent recommendations rather than strict requirements, promoting best practices while recognizing implementation challenges. Intermediate enforcement approaches that state expectations without systematic verification appear most susceptible to policy-practice disconnects. Given substantial investments in peer review, estimated at 100 million hours annually in 2020, editorial policies should be designed to maximize their impact on research quality, ensuring that requirements imposed on authors and reviewers produce measurable benefits commensurate with the time invested [21]. 

This study has several limitations. First, although we included all original research from five high-impact ophthalmology journals, our sample does not represent all ophthalmology publications or non-ophthalmology journals that publish ophthalmic research. Second, the cross-sectional design precludes causal inferences about the relationships between enforcement, guideline use, and manuscript quality. Third, advisable use was determined by one author with expertise in bibliometrics; while this assessment followed explicit criteria matching study designs with guideline definitions, the potential for slight overestimation or underestimation cannot be excluded. Fourth, we assessed only whether guidelines were declared, not the quality of adherence to guideline recommendations.

In conclusion, reporting guideline use in ophthalmology remains low (28.6%), though higher than other specialties. The gap between editorial endorsement and author implementation reveals two opportunities: First, robust evidence determining whether guidelines improve research quality beyond completeness would provide clearer rationale for adoption. Second, editorial policies may benefit from closer alignment with enforcement practices through either systematic implementation with verification or transparent communication that guidelines are recommendations. Bridging this policy-practice gap requires clarifying editorial expectations and implementing feasible enforcement strategies.

## Data Availability

The datasets generated and analyzed during the current study are available from the corresponding author on reasonable request. All data were derived from publicly available journal articles and journal instructions for authors.
